# *CACNA1A*-p.Thr501Met mutation associated with familial hemiplegic migraine: a family report

**DOI:** 10.1186/s10194-021-01297-5

**Published:** 2021-07-28

**Authors:** Marina Romozzi, Guido Primiano, Eleonora Rollo, Lorena Travaglini, Paolo Calabresi, Serenella Servidei, Catello Vollono

**Affiliations:** 1 Dipartimento Universitario di Neuroscienze, Università Cattolica del Sacro Cuore, Rome, Italy; 2 Dipartimento di Scienze dell’invecchiamento, Neurologia, Neurologiche, Ortopediche e della Testa-Collo, Fondazione Policlinico Universitario Agostino Gemelli IRCCS, Rome, Italy; 3 Dipartimento di Scienze dell’invecchiamento, Neurofisiopatologia, Neurologiche, Ortopediche e della Testa-Collo, Fondazione Policlinico Universitario Agostino Gemelli IRCCS, Largo Agostino Gemelli n° 8 -, 00168 Rome, Italy; 4 Dipartimento di Neuroscienze e Neuroriabilitazione, Unità di Malattie Neuromuscolari e Neurodegenerative, IRCCS Ospedale Pediatrico Bambino Gesù, Rome, Italy

**Keywords:** Hemiplegic migraine, *CACNA1A*-p.Thr501Met mutation, Cortical spreading depression, Episodic ataxia, Cerebellar atrophy

## Abstract

**Background and aims:**

Hemiplegic migraine (HM) is a rare form of migraine characterized by the presence of a motor and other types of aura. HM can be sporadic or familial. Familial hemiplegic migraine (FHM) is an autosomal dominant disorder, classified into 3 subtypes, based on the gene involved (*CACNA1A* in FHM1, *ATP1A2* in FHM2 and *SCN1A* in FHM3). The clinical presentation is highly heterogeneous and some attacks may be severe.

We report the clinical characteristics and genetic analysis of 12 patients belonging to a family with *CACNA1A*-p.Thr501Met gene mutation.

**Methods:**

We screened for mutations in *CACNA1A* gene 15 patients belonging to the same family. The exonic sequences of *CACNA1A* were analyzed using a Tru-seq® Custom Amplicon (TSCA) (Illumina Inc., San Diego, CA) targeted capture and paired end library kit. Sanger sequencing was used to confirm *CACNA1A* variants and segregation analysis.

**Results:**

*CACNA1A*-p.Thr501Met mutation was found in 12 of the 15 patients screened, which was compatible with the diagnosis of FHM1.

Attacks of hemiplegic migraine were reported by 10 of the 12 subjects (83.33%). Only one subject developed persistent mild cerebellar symptoms and none of the subjects developed cerebellar atrophy.

**Discussion:**

The variant p.Thr501Met was described previously in association with episodic ataxia and rarely with FHM related to cerebellar symptoms. FHM1 has a broad clinical spectrum and about half of the families have cerebellar involvement. In our study, only one patient developed persistent cerebellar deficits.

These data suggest that *CACNA1A*-p.Thr501Met mutation can occur prevalently as hemiplegic migraine.

**Supplementary Information:**

The online version contains supplementary material available at 10.1186/s10194-021-01297-5.

## Introduction

Hemiplegic migraine (HM) is a rare form of migraine characterized by the presence of a motor aura and other types of aura. HM can occur as a sporadic or familial disorder. Familial hemiplegic migraine (FHM) is an autosomal dominant disease with reduced penetrance and variable expressivity. According to the International Classification of Headache Disorders-3 (ICHD-3), the disease is classified into 3 subtypes, based on the gene involved. Mutations in the *CACNA1A* gene, which encodes the alpha-1A subunit of the P/Q type calcium channel, cause FHM1 [1]. In addition to FHM1, *CACNA1A* gene mutations have been associated with episodic ataxia type 2 and spinocerebellar ataxia type 6 [2, 3]. Mutations in the *ATP1A2* gene, which encodes a catalytic subunit of a sodium/potassium ATPase, cause FHM2. FHM3 is caused by mutations in the *SCN1A* gene that encodes the alpha subunit of the neuronal voltage-gated sodium channel [1]. Recently, a fourth gene, *PRRT2*, which encodes a proline-rich transmembrane protein, has been associated with FHM4 [4]. Therefore, it is likely that the established mutations account for a small percentage of cases of FHM and other not yet identified genes are involved [4].

The clinical presentation of the various forms of FHM is highly variable. Severe attacks can be accompanied by seizures, coma, encephalopathy, fever, cerebellar involvement, cerebral edema or cerebral infarction [5].

The pathophysiological mechanism underlying the motor aura is probably cortical spreading depression (CSD). Mutations related to HM increase neuronal excitability, leading to a higher susceptibility to CSD [6].

Initial therapy with verapamil, flunarizine or acetazolamide is recommended for patients with HM as a preventive treatment. In patients who have persistent aura symptoms that predominate over headache, lamotrigine represents another therapeutic option [5].

About half of the families with FHM1 have cerebellar involvement, including ataxia, gaze-evoked nystagmus, and vermian atrophy [7].

The p.Thr501Met variant was previously described only in episodic ataxia [8] or rarely in HM strictly associated with cerebellar symptoms [9].

We report the clinical characteristics and genetic analysis of 12 patients belonging to a large family with *CACNA1A*-p.Thr501Met gene mutation and a prevalent hemiplegic phenotype.

## Methods

**Fig. 1:**
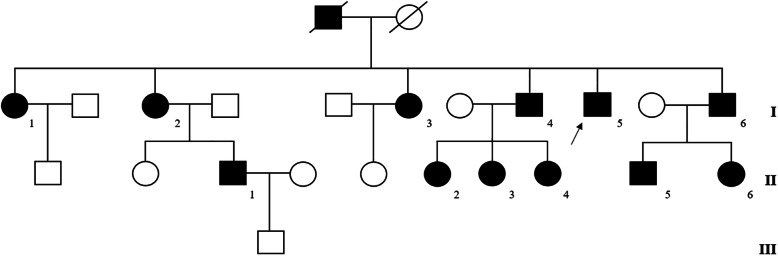
FHM1 pedigree chart of the family. Circles represent the females and squares represent males. Black squares and circles indicate affected males and females, respectively. White squares or circles represent members without FHM. The diagonal lines represent deceased subjects. The arrow indicates the index patient

Upon informed consent, we screened for mutations in *CACNA1A* gene 15 subjects (including unaffected relatives) belonging to the family from 3 successive generations (Fig. 1). The exonic sequences of *CACNA1A* were analyzed using a Tru-seq® Custom Amplicon (TSCA) (Illumina Inc., San Diego, CA) targeted capture and paired end library kit. Variants were detected by means of the HaplotypeCaller software package of the Genome Analysis Toolkit (GATK) suite and filtered so that to include only variants covered by at least 20 reads. High-quality variants were annotated and considered when not reported or having a low allele frequency < 0.005 in genome AD exomes database and occurring with a frequency < 0.01 in our in-house database. Sanger sequencing was used to confirm *CACNA1A* variants and segregation analysis.

## Case presentation

The index patient (Table 1, I-5) is a 71-year-old man who was admitted to our Institute for an episode of migraine associated with fever, right hemiparesis, aphasia and impaired consciousness of a 2-day duration. His past medical history was remarkable for type 2 diabetes, hypertension and Chronic Obstructive Pulmonary Disease (COPD). The patient has been suffering from migraine since the age of 6 years, with a mean frequency of 1 episode per month. Some of his attacks were accompanied by hemiparesis, hemisensory loss, aphasia, confusion, nausea and vomiting, with the symptoms lasting for hours to days. His previous frequency of hemiplegic attacks was of three per year of variable severity. Neurologic examination in between attacks was normal.

**Table 1:** Demographic, clinical characteristics and current prophylactic treatment of the 12 patients with the *CACNA1A*-p.Thr501Met mutation

	Sex	Headache types IHS criteria	Age	Age of onset (years)	Attacks per month	Duration (hours)	Triggers	Comorbidities	Prophylactic Treatment
**I-1**	F	HM	76	24	3	0,5–4	Stress	breast cancer, DM 2	–
**I-2**	F	HM	63	18	2	3–700	Pregnancy	DM 2, AH, panic disorder	–
**I-3**	F	HM	75	15	3	0,5–168	Menstruations	major depressive disorder, DM 2	Amitriptyline
**I-4**	M	HM	68	12	1	4–96	Mild head trauma	AH, DM 2	–
**I-5**	M	HM	71	6	0	3–168	Stress, bright ligths	AH, DM 2, COPD	Acetazolamide + Lamotrigine
**I-6**	M	HM	66	6	2	3–168	–	DM 2, hepatitis B, hiatal hernia	Lamotrigine
**II-1**	M	ETTH	45	15	0	1–48	–	–	–
**II-2**	F	MO	29	20	1	0,5–6	Stress, sleep	–	–
**II-3**	F	HM	24	14	1	12–72	Sleep deprivation, physical exertion	–	–
**II-4**	F	HM	31	16	3	0,5–48	Menstruations	–	–
**II-5**	M	HM	35	15	2	0,5–48	–	thrombophilia	–
**II-6**	F	HM	22	12	3	1–72	Alcohol intake, menstruations	–	–

Abbreviations: *IHS* criteria, International Headache Society criteria; HM, hemiplegic migraine; *ETTH* episodic tension type headache; *MO* migraine without aura; *DM 2* diabetes mellitus type 2; *AH* arterial hypertension; *COPD* Chronic Obstructive Pulmonary Disease

**Fig. 2:**
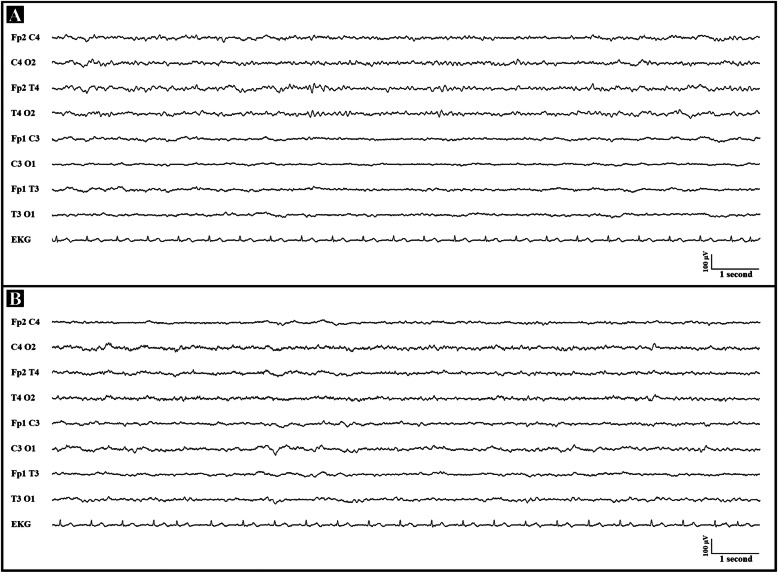
Electroencephalogram findings. Panel **A**: fifteen seconds of ictal EEG: clear amplitude asymmetry of background activity consisting in diffuse depression in left hemispheric fields without epileptiform abnormalities. Panel **B**: fifteen seconds of normal interictal EEG

At the time of our observation, the neurological examination showed right hemiparesis, right Babinski sign and asymmetric pupils (right > left). Cerebrospinal fluid profile was normal. The EEG showed a marked amplitude asymmetry of the background activity consisting in diffuse depression in left hemispheric fields without epileptiform abnormalities (Fig. 2, panel A). Brain CT scan and CT Angiography excluded acute cerebrovascular events. A further brain CT scan performed after 5 days confirmed the absence of acute ischemic or hemorrhagic lesions. Brain MRI could not be performed due to the incompatibility of a metallic pelvic device. Single-Photon Emission Computed Tomography (SPECT) was normal. A complete cardiologic assessment was unremarkable. Lamotrigine up to 100 mg/day and acetazolamide 500 mg/day were prescribed as a preventive treatment.

During hospitalization, the right hemiparesis and aphasia completely resolved. A further EEG performed a week later (Fig. 2, panel B) was normal, showing symmetric background activity. In the next days, the patient developed a cerebellar syndrome with generalized hypotonia, gait ataxia and bilateral dysmetria. Moreover, during the hospitalization he had episodes of psychomotor agitation with visual hallucinations which improved with quetiapine. The patient was discharged and attended a neurorehabilitation program. He did not experience any further episodes of migraine, whereas cerebellar deficits remained stable even at a 18-month follow-up.

A further brain CT scan performed after 5 months did not show significant cerebellar atrophy.

### Family history

The family history was remarkable for headache, with several cases of hemiplegic migraine.

Patient I-1 (Fig. 1, I-1) since the age of 25 years had suffered from recurrent attacks of migraine without aura (MO). She reported a single hemiplegic episode at the age of 30 years.

Patient I-2 (Fig. 1, I-2) has been having attacks of migraine with typical aura and experienced several hemiplegic attacks lasting up to days.

Patient I-3 (Fig. 1, I-3) has been suffering from migraine with aura and hemiplegic attacks since her early childhood. Since the age of 30, she has been free from attacks with motor aura.

Patient I-4 (Fig. 1, I-4) had frequent attacks of HM followed by drowsiness since the age of 12. He had 10 severe long-lasting attacks which required hospitalization.

Patient I-6 (Fig. 1, I-6) has been having hemiplegic migraine since the age of 5. He had multiple episodes of migraine associated with hemiparesis, sensory deficits, aphasia, diminished consciousness, fever and psychiatric symptoms with formed hallucinations, lasting up to 7 days.

Patient II-1 (Fig. 1, II-1) never suffered from hemiplegic attacks, but he was diagnosed with episodic tension-type headache (ETTH). Patients II-3, II-4, II-5 and II-6 (Fig. 1, II-3, II-4, II-5 and II-6) had classic HM and patient II-2 was diagnosed with MO.

The index patient’s father, not available for genetic analysis, had transient episodes of limb weakness followed by headache in his young adulthood.

## Results

Analysis of *CACNA1A* revealed Thr501Met mutation in 12 of the 15 subjects screened, which was compatible with the diagnosis of FHM1. The 12 cases were 7 women (58.33%) and 5 men (41.67%). Mean age of onset was 14.4 ± 5.2 (range 6–24) and the mean age at examination was 50.4 ± 21.3 (range 22–76).

Clinical characteristics and current prophylactic treatment of the 12 cases carrying *CACNA1A* mutation are summarized in Table 1.

Attacks of hemiplegic migraine that fulfilled the criteria of the ICHD-3 [1] were reported by 10 of the 12 subjects (83.33%). The mean monthly migraine days in patients with HM was 2 ± 1.

All subjects affected by HM also reported attacks of migraine with non-hemiplegic aura and MO. One subject carrying p.Thr501Met mutation (8.33%) was diagnosed with MO and one subject (8.33%) with ETTH. Emotional stress, menstruations and sleep deprivation were the most frequent triggering factors.

None of the subjects, except for the index patient (Fig. 1, I-5), had ever experienced cerebellar symptoms.

A brain MRI scan was performed in 9 patients carrying *CACNA1A* mutation showing no cerebellar atrophy.

### Discussion

We described a large family with *CACNA1A*-p.Thr501Met mutation whose prevalent clinical expression was hemiplegic migraine. The heterozygous missense variant p.Thr501Met was described previously in association with episodic ataxia [8] and in one patient with FHM associated to cerebellar symptoms [9]. This mutation changes a hydrophilic amino acid versus a hydrophobic one, probably altering the characteristic transmembrane conformation of the P/Q type calcium channel subunit [8, 9]. P/Q type calcium channels are expressed in the brain and particularly in cerebellar Purkinje and granule cells. Different FHM mutations induce profound Purkinje cell dysfunctions finally leading to neuronal loss and cerebellar atrophy [6]. FHM1 has a broad clinical spectrum and more than half of the families have cerebellar involvement [7].

The index patient had a previous history of recurrent long-lasting hemiplegic migraine attacks sometimes associated with reduced consciousness, fever and psychiatric symptoms. The EEG left hemispheric depression during the first acute phase of the attack represents the neurophysiological correlate of the reversible cortical inhibition. Furthermore, the patient developed a cerebellar syndrome with persistent functional impairment. Acetazolamide and lamotrigine were effective in preventing further attacks.

Both hemiplegia and cerebellar involvement find an adequate explanation with the disfunction of the P/Q type calcium channels subunit. Instead, the psychiatric manifestations are more difficult to justify. Hallucinations are commonly reported as a FHM acute presentation associated with migraine attacks [10, 11]. Another possible explanation for the index patient’s symptoms could be the occurrence of delirium in an elderly hospitalized patient. However, the patient had no relevant predisposing risk factors for delirium, other than old age, nor did he experience alcohol withdrawal, use of psychotropic medications, sleep deprivation, infections [12]. Furthermore, the psychiatric manifestation are consistent with the involvement of ion channels in other neuropsychiatric disorders [13]. In this view, the complexity of the clinical manifestations of our index patient confirms the crucial role of calcium voltage-gated channel subunit alpha-1A in CNS function.

None of the 12 patients of our family had permanent motor, sensory, language, visual symptoms except for the index patient who developed persistent cerebellar deficits.

Furthermore, in our family there was a remarkable clinical heterogeneity which could be explained by the variable penetrance of the *CACNA1A* mutations being the asymptomatic or mildly symptomatic cases mostly from the second generation [7, 14].

## Conclusion

To conclude, the *CACNA1A*-p.Thr501Met mutation is associated with distinct phenotypes, ranging from cerebellar to hemiplegic syndromes. Our data suggest that *CACNA1A*-p.Thr501Met mutation can occur prevalently as hemiplegic migraine. However, it is not clear why some *CACNA1A* mutations cause a prevalent hemiplegic pattern, others a prevalent ataxic phenotype and others a complex mixed pattern.

## Supplementary Information

**Additional file 1.**

## Data Availability

no statistical analysis was conducted.
