# Supplementary material for: CACNA1A-p.Thr501Met mutation associated with familial hemiplegic migraine: a family report
Source: J Headache Pain. 2021 Jul 28;22(1):85. doi: 10.1186/s10194-021-01297-5 (PMC8317284; doi:10.1186/s10194-021-01297-5)
Supplement: Supplementary file 1 — Additional file 1. [file 10194_2021_1297_MOESM1_ESM.docx]

**Patient I-1** (Female, 76 years old)

Since the age of 25 years, she has suffered from recurrent attacks of migraine associated with strong photophobia, phonophobia, nausea and vomiting with an average frequency of 1-2 per month. She reported a single hemiplegic episode at the age of thirty years which lasted for about two hours and required hospitalization.

The patient has a past history of breast cancer. She also suffers from type 2 diabetes. Neurological examination was unrevealing. She is currently not taking any prophylactic therapy. She takes nonsteroidal anti-inflammatory drugs (NSAIDs) as a symptomatic treatment.

**Patient I-2** (Female, 63 years old)

The patient has been having attacks of migraine with aura since the first months after childbirth, at the age of eighteen years. Some of her migraine attacks were accompanied by hemiparesis, hemisensory loss and aphasia with the symptoms lasting for about thirty minutes to hours, followed by a full recovery. In particular two episode resulted in a prolonged deficit requiring hospitalization. The current frequency of attacks is once every month. Her past medical history was significant for type 2 diabetes and hypertension. She also suffers from recurrent panic attacks. Neurological examination revealed pronation of the right arm and drift of the right leg. She did not undergo regular treatment except for symptomatic therapy.

**Patient I-3** (Female, 75 years old)

The patient has been suffering from migraine with aura since her early childhood. Aura consists of motor symptoms (weakness in one arm), sensory and dysphasic manifestations and can last from hours to seven days. The first hemiplegic attack happened during the third trimester of pregnancy and it was accompanied by speech difficulties. Headache attacks were associated with nausea and vomiting. She reported an improvement in severity of the attacks in adulthood, while the frequency remained constant with an average of 3 per month. Attacks with motor symptoms suspended at the age of thirty.

Hemiplegia typically occurred on the same side during recurrent attacks, though side switching between attacks was reported. The patient was diagnosed with major depressive disorder. The patient also suffers from daily chronic tension-type headache and type 2 diabetes. Neurological and general physical examinations were normal. Amitryptiline was prescribed as a prophylactic medication.

**Patient I-4** (Male, 68 years old)

The patient first developed symptoms at the age of twelve years. He presented numbness and a paralysis of the right side, accompanied by strong photophobia, phonophobia, nausea and vomiting and followed by headache. Similar attacks occurred over the following years. The frequency of the attacks decreased from one every week to once a month. He remembers ten severe attacks which required hospitalization. During severe attacks motor symptoms could last up to one week. His past medical history was consistent for hypertension and type 2 diabetes. Neurological examination was unremarkable. He did not undergo regular treatment except for simple analgesics.

**Patient I-5 (index case)**

**Patient I-6** (Male, 66 years old)

The patient has been having hemiplegic migraine since the age of five years. He had multiple episodes of migraine associated with hemiparesis, paraesthesia, prolonged somnolence, aphasia, confusion, diminished consciousness and fever. Reported visual aura symptoms included scotomas, formed hallucinations (such as bucolic farm scene and birds flying) and dysmetropsi (“Alice in Wonderland Syndrome”).

Associated symptoms were photophobia, phonophobia, nausea and vomiting. He remembers more than ten severe attacks with hospitalizations. The frequency of episodes is highly variable, ranging from one to four per month. Each episode lasted from 3-4 hours to five days and was characterized by a progressive reduction of symptoms and a full recovery.

MRI of the brain performed in the acute phase demonstrated non-specific hyperintense foci on T2-weighted images. A SPECT performed during the hemiplegic attack was consistent with diffuse hyperemia of the contralateral hemisphere to the hemiparesis.

The medical history was significant for type 2 diabetes, hepatitis B and hiatal hernia.

Neurological examination in between the attacks was unremarkable and no cerebellar signs were present.

In the past prophylactic therapies with beta blockers, antiepileptic drugs such as sodium valproate and topiramate, calcium channel blockers, acetazolamide and corticosteroids were prescribed but they were not effective. Lamotrigine prophylaxis is current ongoing.

**Patient II-1** (Male, 45 years old)

The patient has been suffering from migraine since he was fifteen years old. His episodes are characterized by pressing tightening pain of moderate intensity, located in the left side, usually lasting from 1-2 hours to 48 hours and responsive to common analgesics. He never suffered from hemiplegic episodes. He experiences attacks about once a month. The patient rated the pain 1/10 on the visual analog scale. The medical history was unremarkable except for sciatica in treatment with pregabalin. Neurological examination was normal.

**Patient II-2** (Female, 29 years old)

The patient has been suffering from migraine without aura since the age of twenty. Episodes are characterized by unilateral moderate pulsatile headache usually originated in the right temple, without any other symptom. The pain is responsive to simple analgesics. In general, symptoms lasted 30 minutes to 3-4 hours and occurred about once per month. Neurological examination and past medical history were unremarkable.

**Patient II-3** (Female, 24 years old)

The patient first developed symptoms at 14 years of age. She has been suffering from classical hemiplegic migraine with motor, sensory and dysphasic aura, accompanied by photophobia, nausea and vomiting.

She had had experienced a prolonged episode of hemiparesis and hemisensory loss with confusion and difficulty in speaking. In all other episodes, he would recover spontaneously and completely within 72 hours. Usually, those episodes subsided with paracetamol or NSAIDs. Neurological examination and past medical history were unremarkable.

**Patient II-4** (Female, 31 years old)

The patient experienced her first symptoms at the age of sixteen years old. She had her first hemiplegic attack the age of nineteen years, when she experienced an aura with visual disturbances, confusion and weakness of her right upper and lower limbs along with numbness and tingling of either side and slurring of speech, follow by intense temporal headache, vomiting, photophobia and phonofobia. Lateralization of signs and symptoms varied little with each episode usually resulting in right hemiplegia. Since then, about ten similar attacks occurred in the following years and lasted for 24-48 hours. The duration of the neurologic deficit averaged 2 hours, resolving to severe headache, nausea, and vomiting. These episodes were responsive to oral triptans.

At 20 years of age, the patient experienced an episode that resulted in a prolonged deficit requiring hospitalization. MRI scan performed at that time was normal. She also suffers from migraine without aura with a perimenstrual pattern of attacks. Neurological examination and past medical history were unremarkable.

**Patient II-5** (Male, 35 years old)

The patient had multiple episodes of migraine associated with hemiparesis, paraesthesia alternating with right and left sides, prolonged somnolence, aphasia and confusion, since the age of fifteen years, which could last up to two days, accompanied by photophobia and phonofobia, of favorable spontaneous resolution. The average frequency of the attacks is 1-2 per month. The headache was partly relieved with NSAIDs. Therefore, during a severe migraine attack he required hospitalization. He also suffers from thrombophilia and he is current on anticoagulation therapy with coumadin. Neurological examination was normal.

**Patient II-6** (Female, 22 years old)

The patient had been suffering from migraine with aura since the age of twelve. The attacks were accompanied by hemiparesis, aphasia, sensory disturbances lasting for few hours and then reverting back to normalcy preceded always with unilateral, throbbing headache associated with nausea, photophobia, phonophobia and osmophobia. Her last hemiplegic attack occurred when the patient was seventeen years old, while migraine with sensory and dysphasic manifestations still happens frequently.

Topiramate was prescribed as a prophylactic therapy but it was not effective, so the patient stopped the treatment. Neurological examination was normal.
